# Supplementary material for: Association of Vitamin D Prescribing and Clinical Outcomes in Adults Hospitalized with COVID-19
Source: Nutrients. 2022 Jul 26;14(15):3073. doi: 10.3390/nu14153073 (PMC9332080; doi:10.3390/nu14153073)
Supplement: Supplementary file 1 [file nutrients-14-03073-s001.zip › nutrients-1806680-supplementary.pdf]

Supplemetary

Supplementary Table S1: Detail of Characteristics of Patients Associated with Key Outcomes in Hospitalized Patients with COVID-19

	Overall	Death/Hospice[2]	LOS > 5 days	Mechanical Ventilation/ECMO
	N=158,835	*N=16,838 (11%)*[1]	*N=75,751 (48%)*[1]	*N=20,417 (13%)*[1]
Age at Diagnosis				
18-29	12165 (7.7%)	131 (1.1%)	2928 (24%)	741 (6.1%)
30-49	32808 (21%)	1058 (3.2%)	11889 (36%)	3351 (10%)
50-64	43850 (28%)	3544 (8.1%)	21783 (50%)	6783 (15%)
65-74	31991 (20%)	4271 (13%)	17579 (55%)	5355 (17%)
75 and older	38021 (24%)	7834 (21%)	21572 (57%)	4187 (11%)
Sex				
Female	77886 (49%)	6781 (8.7%)	33409 (43%)	7668 (9.8%)
Male	79996 (50%)	10055 (13%)	41841 (52%)	12702 (16%)
Race				
White	83362 (52%)	9444 (11%)	39337 (47%)	10131 (12%)
Black or African American	30711 (19%)	2946 (9.6%)	15268 (50%)	4192 (14%)
Asian	6210 (3.9%)	716 (12%)	3150 (51%)	909 (15%)
Other	4891 (3.1%)	418 (8.5%)	2512 (51%)	842 (17%)
Unknown	33661 (21%)	3314 (9.8%)	15484 (46%)	4343 (13%)
Ethnicity				
Hispanic or Latino	33151 (21%)	3048 (9.2%)	14985 (45%)	4450 (13%)
Not Hispanic or Latino	107674 (68%)	11942 (11%)	52383 (49%)	13397 (12%)
Unknown	18010 (11%)	1848 (10%)	8383 (47%)	2570 (14%)
Quarter of Diagnosis				
2020 Q1	7890 (5.0%)	1450 (18%)	4809 (61%)	2024 (26%)
2020 Q2	32939 (21%)	4455 (14%)	17216 (52%)	4947 (15%)
2020 Q3	17277 (11%)	1330 (7.7%)	7542 (44%)	1992 (12%)
2020 Q4	47084 (30%)	4917 (10%)	22039 (47%)	5311 (11%)
2021 Q1	35179 (22%)	3295 (9.4%)	16211 (46%)	3843 (11%)
2021 Q2	14204 (8.9%)	999 (7.0%)	5965 (42%)	1702 (12%)
2021 Q3	4262 (2.7%)	392 (9.2%)	1969 (46%)	598 (14%)
RUCA Category: Patient residence				
Urban	102191 (64%)	11589 (11%)	49563 (49%)	13760 (13%)
Large rural	7969 (5.0%)	1218 (15%)	4135 (52%)	1603 (20%)
Small rural	4010 (2.5%)	629 (16%)	2184 (54%)	764 (19%)
Isolated	2274 (1.4%)	344 (15%)	1257 (55%)	431 (19%)
Unknown	42391 (27%)	3058 (7.2%)	18612 (44%)	3859 (9.1%)

Reported BMI				
Obese	48539 (31%)	4766 (9.8%)	23255 (48%)	7967 (16%)
Overweight	29421 (19%)	3303 (11%)	14098 (48%)	3938 (13%)
Normal weight	21199 (13%)	2813 (13%)	10793 (51%)	2430 (11%)
Underweight	3569 (2.2%)	385 (11%)	1872 (52%)	223 (6.2%)
Unknown	56107 (35%)	5571 (9.9%)	25733 (46%)	5859 (10%)
Charlson Index Category				
0	49171 (31%)	3398 (6.9%)	19020 (39%)	4540 (9.2%)
1	18967 (12%)	1598 (8.4%)	8354 (44%)	2038 (11%)
2	14140 (8.9%)	1350 (9.5%)	6848 (48%)	1566 (11%)
3 or more	41074 (26%)	5622 (14%)	22117 (54%)	5376 (13%)
Unknown	35483 (22%)	4870 (14%)	19412 (55%)	6897 (19%)
Vitamin D treatment				
No	129842 (82%)	13185 (10%)	58372 (45%)	15520 (12%)
Yes	28993 (18%)	3653 (13%)	17379 (60%)	4897 (17%)
Died	16838 (11%)	NA	12566 (75%)	8982 (53%)
Length of stay > 5 days	75751 (48%)	12566 (17%)	NA	17480 (23%)
Mechanical Ventilation/ECMO	20417 (13%)	8982 (44%)	17480 (86%)	NA

1. Statistic presented: n (%). Percent = column percent
2. Death or referral to hospice during hospitalization

Supplementary Table S2: Detail of Characteristics of Patients Associated with Key Outcomes in Hospitalized Patients with Severe COVID-19

	Overall	Death/Hospice	LOS > 5 Days	Mechanical Ventilation/ECMO
	N=81,381	*N=16,838 (21%)*	*N=75,751 (93%)*	*N=20,417 (25%)*
Age at Diagnosis				
18-29	3074 (3.8%)	131 (4.3%)	2928 (95%)	741 (24%)
30-49	12376 (15%)	1058 (8.5%)	11889 (96%)	3351 (27%)
50-64	22820 (28%)	3544 (16%)	21783 (95%)	6783 (30%)
65-74	18779 (23%)	4271 (23%)	17579 (94%)	5355 (29%)
75 and older	24332 (30%)	7834 (32%)	21572 (89%)	4187 (17%)
Sex				
Female	35897 (44%)	6781 (19%)	33409 (93%)	7668 (21%)
Male	44979 (55%)	10055 (22%)	41841 (93%)	12702 (28%)
Race				
White	42543 (52%)	9444 (22%)	39337 (92%)	10131 (24%)
Black or African American	16266 (20%)	2946 (18%)	15268 (94%)	4192 (26%)
Asian	3368 (4.1%)	716 (21%)	3150 (94%)	909 (27%)
Other	2617 (3.2%)	418 (16%)	2512 (96%)	842 (32%)
Unknown	16587 (20%)	3314 (20%)	15484 (93%)	4343 (26%)
Ethnicity				
Hispanic or Latino	15841 (19%)	3048 (19%)	14985 (95%)	4450 (28%)
Not Hispanic or Latino	56412 (69%)	11942 (21%)	52383 (93%)	13397 (24%)
Unknown	9128 (11%)	1848 (20%)	8383 (92%)	2570 (28%)
Quarter of Diagnosis				
2020 Q1	5290 (6.5%)	1450 (27%)	4809 (91%)	2024 (38%)
2020 Q2	18918 (23%)	4455 (24%)	17216 (91%)	4947 (26%)
2020 Q3	7987 (9.8%)	1330 (17%)	7542 (94%)	1992 (25%)
2020 Q4	23484 (29%)	4917 (21%)	22039 (94%)	5311 (23%)
2021 Q1	17282 (21%)	3295 (19%)	16211 (94%)	3843 (22%)
2021 Q2	6356 (7.8%)	999 (16%)	5965 (94%)	1702 (27%)
2021 Q3	2064 (2.5%)	392 (19%)	1969 (95%)	598 (29%)
RUCA Category: Patient residence				
Urban	53421 (66%)	11589 (22%)	49563 (93%)	13760 (26%)
Large rural	4531 (5.6%)	1218 (27%)	4135 (91%)	1603 (35%)
Small rural	2388 (2.9%)	629 (26%)	2184 (91%)	764 (32%)
Isolated	1382 (1.7%)	344 (25%)	1257 (91%)	431 (31%)
Unknown	19659 (24%)	3058 (16%)	18612 (95%)	3859 (20%)
Reported BMI				

Obese	24622 (30%)	4766 (19%)	23255 (94%)	7967 (32%)
Overweight	15099 (19%)	3303 (22%)	14098 (93%)	3938 (26%)
Normal weight	11758 (14%)	2813 (24%)	10793 (92%)	2430 (21%)
Underweight	2010 (2.5%)	385 (19%)	1872 (93%)	223 (11%)
Unknown	27892 (34%)	5571 (20%)	25733 (92%)	5859 (21%)
Charlson Index Category				
0	20140 (25%)	3398 (17%)	19020 (94%)	4540 (23%)
1	8918 (11%)	1598 (18%)	8354 (94%)	2038 (23%)
2	7285 (9.0%)	1350 (19%)	6848 (94%)	1566 (21%)
3 or more	24098 (30%)	5622 (23%)	22117 (92%)	5376 (22%)
Unknown	20940 (26%)	4870 (23%)	19412 (93%)	6897 (33%)
Vitamin D treatment				
No	63249 (78%)	13185 (21%)	58372 (92%)	15520 (25%)
Yes	18132 (22%)	3653 (20%)	17379 (96%)	4897 (27%)
Died	16838 (21%)	NA	12566 (75%)	8982 (53%)
Length of stay > 5 days	75751 (93%)	12566 (17%)	NA	17480 (23%)
Mechanical Ventilation/ECMO	20417 (25%)	8982 (44%)	17480 (86%)	NA

Statistic presented: n (%). Percent = column percent

Supplementary Table S3: Full Detail of Characteristics of Patients Associated with Key Outcomes in Hospitalized Patients with COVID-19 (n=158,835)

	Overall	Death/Referral to hospice		Length of Stay > 5 Days		Mechanical Vent/ECMO	
		Survived	Died	LOS <=5 days	LOS > 5 days	No	Yes
	N=158,835	*N=141,997 (89%)*	*N=16,838 (11%)*	*N=83,084 (52%)*	*N=75,751 (48%)*	*N=138,418 (87%)*	*N=20,417 (13%)*
Age at Diagnosis							
18-29	12165 (7.7%)	12034 (99%)	131 (1.1%)	9237 (76%)	2928 (24%)	11424 (94%)	741 (6.1%)
30-49	32808 (21%)	31750 (97%)	1058 (3.2%)	20919 (64%)	11889 (36%)	29457 (90%)	3351 (10%)
50-64	43850 (28%)	40306 (92%)	3544 (8.1%)	22067 (50%)	21783 (50%)	37067 (85%)	6783 (15%)
65-74	31991 (20%)	27720 (87%)	4271 (13%)	14412 (45%)	17579 (55%)	26636 (83%)	5355 (17%)
75 and older	38021 (24%)	30187 (79%)	7834 (21%)	16449 (43%)	21572 (57%)	33834 (89%)	4187 (11%)
Sex							
Female	77886 (49%)	71105 (91%)	6781 (8.7%)	44477 (57%)	33409 (43%)	70218 (90%)	7668 (9.8%)
Male	79996 (50%)	69941 (87%)	10055 (13%)	38155 (48%)	41841 (52%)	67294 (84%)	12702 (16%)
Race							
White	83362 (52%)	73918 (89%)	9444 (11%)	44025 (53%)	39337 (47%)	73231 (88%)	10131 (12%)
Black or African American	30711 (19%)	27765 (90%)	2946 (9.6%)	15443 (50%)	15268 (50%)	26519 (86%)	4192 (14%)
Asian	6210 (3.9%)	5494 (88%)	716 (12%)	3060 (49%)	3150 (51%)	5301 (85%)	909 (15%)
Other	4891 (3.1%)	4473 (91%)	418 (8.5%)	2379 (49%)	2512 (51%)	4049 (83%)	842 (17%)
Unknown	33661 (21%)	30347 (90%)	3314 (9.8%)	18177 (54%)	15484 (46%)	29318 (87%)	4343 (13%)
Ethnicity							
Hispanic or Latino	33151 (21%)	30103 (91%)	3048 (9.2%)	18166 (55%)	14985 (45%)	28701 (87%)	4450 (13%)
Not Hispanic or Latino	107674 (68%)	95732 (89%)	11942 (11%)	55291 (51%)	52383 (49%)	94277 (88%)	13397 (12%)
Unknown	18010 (11%)	16162 (90%)	1848 (10%)	9627 (53%)	8383 (47%)	15440 (86%)	2570 (14%)
Quarter of Diagnosis							
2020 Q1	7890 (5.0%)	6440 (82%)	1450 (18%)	3081 (39%)	4809 (61%)	5866 (74%)	2024 (26%)
2020 Q2	32939 (21%)	28484 (86%)	4455 (14%)	15723 (48%)	17216 (52%)	27992 (85%)	4947 (15%)
2020 Q3	17277 (11%)	15947 (92%)	1330 (7.7%)	9735 (56%)	7542 (44%)	15285 (88%)	1992 (12%)

2020 Q4	47084 (30%)	42167 (90%)	4917 (10%)	25045 (53%)	22039 (47%)	41773 (89%)	5311 (11%)
2021 Q1	35179 (22%)	31884 (91%)	3295 (9.4%)	18968 (54%)	16211 (46%)	31336 (89%)	3843 (11%)
2021 Q2	14204 (8.9%)	13205 (93%)	999 (7.0%)	8239 (58%)	5965 (42%)	12502 (88%)	1702 (12%)
2021 Q3	4262 (2.7%)	3870 (91%)	392 (9.2%)	2293 (54%)	1969 (46%)	3664 (86%)	598 (14%)
RUCA Category: Patient residence							
Urban	102191 (64%)	90602 (89%)	11589 (11%)	52628 (51%)	49563 (49%)	88431 (87%)	13760 (13%)
Large rural	7969 (5.0%)	6751 (85%)	1218 (15%)	3834 (48%)	4135 (52%)	6366 (80%)	1603 (20%)
Small rural	4010 (2.5%)	3381 (84%)	629 (16%)	1826 (46%)	2184 (54%)	3246 (81%)	764 (19%)
Isolated	2274 (1.4%)	1930 (85%)	344 (15%)	1017 (45%)	1257 (55%)	1843 (81%)	431 (19%)
Unknown	42391 (27%)	39333 (93%)	3058 (7.2%)	23779 (56%)	18612 (44%)	38532 (91%)	3859 (9.1%)
Reported BMI							
Obese	48539 (31%)	43773 (90%)	4766 (9.8%)	25284 (52%)	23255 (48%)	40572 (84%)	7967 (16%)
Overweight	29421 (19%)	26118 (89%)	3303 (11%)	15323 (52%)	14098 (48%)	25483 (87%)	3938 (13%)
Normal weight	21199 (13%)	18386 (87%)	2813 (13%)	10406 (49%)	10793 (51%)	18769 (89%)	2430 (11%)
Underweight	3569 (2.2%)	3184 (89%)	385 (11%)	1697 (48%)	1872 (52%)	3346 (94%)	223 (6.2%)
Unknown	56107 (35%)	50536 (90%)	5571 (9.9%)	30374 (54%)	25733 (46%)	50248 (90%)	5859 (10%)
Charlson Index Category							
0	49171 (31%)	45773 (93%)	3398 (6.9%)	30151 (61%)	19020 (39%)	44631 (91%)	4540 (9.2%)
1	18967 (12%)	17369 (92%)	1598 (8.4%)	10613 (56%)	8354 (44%)	16929 (89%)	2038 (11%)
2	14140 (8.9%)	12790 (90%)	1350 (9.5%)	7292 (52%)	6848 (48%)	12574 (89%)	1566 (11%)
3 or more	41074 (26%)	35452 (86%)	5622 (14%)	18957 (46%)	22117 (54%)	35698 (87%)	5376 (13%)
Unknown	35483 (22%)	30613 (86%)	4870 (14%)	16071 (45%)	19412 (55%)	28586 (81%)	6897 (19%)
Vitamin D treatment							
No	129842 (82%)	116657 (90%)	13185 (10%)	71470 (55%)	58372 (45%)	114322 (88%)	15520 (12%)
Yes	28993 (18%)	25340 (87%)	3653 (13%)	11614 (40%)	17379 (60%)	24096 (83%)	4897 (17%)
Died	16838 (11%)	NA	NA	4272 (25%)	12566 (75%)	7856 (47%)	8982 (53%)
Length of stay > 5 days	75751 (48%)	63185 (83%)	12566 (17%)	NA	NA	58271 (77%)	17480 (23%)
Mechanical Ventilation/ECMO	20417 (13%)	11435 (56%)	8982 (44%)	2937 (14%)	17480 (86%)	NA	NA

Supplementary Table S4: Full Detail of Characteristics Associated with Key Outcomes in Hospitalized Patients with Severe COVID-19 (n=81,381)

	Overall	Death/Referral to hospice		Length of Stay > 5 Days		Mechanical Vent/ECMO	
	Overall	Survived	Died	<=5 days	> 5 days	No	Yes
	N=81,381	*N=64,543 (79%)*	*N=16,838 (21%)*	*N=5630 (7%)*	*N=75,751 (93%)*	*N=60,964 (75%)*	*N=20,417 (25%)*
Age at Diagnosis							
18-29	3074 (3.8%)	2943 (96%)	131 (4.3%)	146 (4.7%)	2928 (95%)	2333 (76%)	741 (24%)
30-49	12376 (15%)	11318 (91%)	1058 (8.5%)	487 (3.9%)	11889 (96%)	9025 (73%)	3351 (27%)
50-64	22820 (28%)	19276 (84%)	3544 (16%)	1037 (4.5%)	21783 (95%)	16037 (70%)	6783 (30%)
65-74	18779 (23%)	14508 (77%)	4271 (23%)	1200 (6.4%)	17579 (94%)	13424 (71%)	5355 (29%)
75 and older	24332 (30%)	16498 (68%)	7834 (32%)	2760 (11%)	21572 (89%)	20145 (83%)	4187 (17%)
Sex							
Female	35897 (44%)	29116 (81%)	6781 (19%)	2488 (6.9%)	33409 (93%)	28229 (79%)	7668 (21%)
Male	44979 (55%)	34924 (78%)	10055 (22%)	3138 (7.0%)	41841 (93%)	32277 (72%)	12702 (28%)
Race							
White	42543 (52%)	33099 (78%)	9444 (22%)	3206 (7.5%)	39337 (92%)	32412 (76%)	10131 (24%)
Black or African American	16266 (20%)	13320 (82%)	2946 (18%)	998 (6.1%)	15268 (94%)	12074 (74%)	4192 (26%)
Asian	3368 (4.1%)	2652 (79%)	716 (21%)	218 (6.5%)	3150 (94%)	2459 (73%)	909 (27%)
Other	2617 (3.2%)	2199 (84%)	418 (16%)	105 (4.0%)	2512 (96%)	1775 (68%)	842 (32%)
Unknown	16587 (20%)	13273 (80%)	3314 (20%)	1103 (6.6%)	15484 (93%)	12244 (74%)	4343 (26%)
Ethnicity							
Hispanic or Latino	15841 (19%)	12793 (81%)	3048 (19%)	856 (5.4%)	14985 (95%)	11391 (72%)	4450 (28%)
Not Hispanic or Latino	56412 (69%)	44470 (79%)	11942 (21%)	4029 (7.1%)	52383 (93%)	43015 (76%)	13397 (24%)
Unknown	9128 (11%)	7280 (80%)	1848 (20%)	745 (8.2%)	8383 (92%)	6558 (72%)	2570 (28%)
Quarter of Diagnosis							
2020 Q1	5290 (6.5%)	3840 (73%)	1450 (27%)	481 (9.1%)	4809 (91%)	3266 (62%)	2024 (38%)
2020 Q2	18918 (23%)	14463 (76%)	4455 (24%)	1702 (9.0%)	17216 (91%)	13971 (74%)	4947 (26%)
2020 Q3	7987 (9.8%)	6657 (83%)	1330 (17%)	445 (5.6%)	7542 (94%)	5995 (75%)	1992 (25%)

2020 Q4	23484 (29%)	18567 (79%)	4917 (21%)	1445 (6.2%)	22039 (94%)	18173 (77%)	5311 (23%)
2021 Q1	17282 (21%)	13987 (81%)	3295 (19%)	1071 (6.2%)	16211 (94%)	13439 (78%)	3843 (22%)
2021 Q2	6356 (7.8%)	5357 (84%)	999 (16%)	391 (6.2%)	5965 (94%)	4654 (73%)	1702 (27%)
2021 Q3	2064 (2.5%)	1672 (81%)	392 (19%)	95 (4.6%)	1969 (95%)	1466 (71%)	598 (29%)
RUCA Category: Patient residence							
Urban	53421 (66%)	41832 (78%)	11589 (22%)	3858 (7.2%)	49563 (93%)	39661 (74%)	13760 (26%)
Large rural	4531 (5.6%)	3313 (73%)	1218 (27%)	396 (8.7%)	4135 (91%)	2928 (65%)	1603 (35%)
Small rural	2388 (2.9%)	1759 (74%)	629 (26%)	204 (8.5%)	2184 (91%)	1624 (68%)	764 (32%)
Isolated	1382 (1.7%)	1038 (75%)	344 (25%)	125 (9.0%)	1257 (91%)	951 (69%)	431 (31%)
Unknown	19659 (24%)	16601 (84%)	3058 (16%)	1047 (5.3%)	18612 (95%)	15800 (80%)	3859 (20%)
Reported BMI							
Obese	24622 (30%)	19856 (81%)	4766 (19%)	1367 (5.6%)	23255 (94%)	16655 (68%)	7967 (32%)
Overweight	15099 (19%)	11796 (78%)	3303 (22%)	1001 (6.6%)	14098 (93%)	11161 (74%)	3938 (26%)
Normal weight	11758 (14%)	8945 (76%)	2813 (24%)	965 (8.2%)	10793 (92%)	9328 (79%)	2430 (21%)
Underweight	2010 (2.5%)	1625 (81%)	385 (19%)	138 (6.9%)	1872 (93%)	1787 (89%)	223 (11%)
Unknown	27892 (34%)	22321 (80%)	5571 (20%)	2159 (7.7%)	25733 (92%)	22033 (79%)	5859 (21%)
Charlson Index Category							
0	20140 (25%)	16742 (83%)	3398 (17%)	1120 (5.6%)	19020 (94%)	15600 (77%)	4540 (23%)
1	8918 (11%)	7320 (82%)	1598 (18%)	564 (6.3%)	8354 (94%)	6880 (77%)	2038 (23%)
2	7285 (9.0%)	5935 (81%)	1350 (19%)	437 (6.0%)	6848 (94%)	5719 (79%)	1566 (21%)
3 or more	24098 (30%)	18476 (77%)	5622 (23%)	1981 (8.2%)	22117 (92%)	18722 (78%)	5376 (22%)
Unknown	20940 (26%)	16070 (77%)	4870 (23%)	1528 (7.3%)	19412 (93%)	14043 (67%)	6897 (33%)
Vitamin D treatment							
No	63249 (78%)	50064 (79%)	13185 (21%)	4877 (7.7%)	58372 (92%)	47729 (75%)	15520 (25%)
Yes	18132 (22%)	14479 (80%)	3653 (20%)	753 (4.2%)	17379 (96%)	13235 (73%)	4897 (27%)
Died	16838 (21%)	NA	NA	4272 (25%)	12566 (75%)	7856 (47%)	8982 (53%)
Length of stay > 5 days	75751 (93%)	63185 (83%)	12566 (17%)	NA	NA	58271 (77%)	17480 (23%)
Mechanical Ventilation/ECMO	20417 (25%)	11435 (56%)	8982 (44%)	2937 (14%)	17480 (86%)	NA	NA

Supplementary Table S5: Multivariable Models for Vitamin D Receipt and Key Outcomes: Full Cohort

	Death/Hospice<br>AOR (CI 95%)	Length of Stay > 5 days<br>AOR (CI 95%)	Mechanical Ventilation/ECMO<br>AOR (CI 95%)
**Age category**			
75 and older	Reference	Reference	Reference
65-74	0.55(0.53- 0.57)	0.90(0.87- 0.93)	1.36(1.30- 1.43)
50-64	0.31(0.29- 0.32)	0.73(0.70- 0.75)	1.14(1.09- 1.19)
30-49	0.12(0.11- 0.13)	0.45(0.43- 0.46)	0.70(0.66- 0.74)
18-29	0.04(0.04- 0.05)	0.28(0.27- 0.30)	0.49(0.45- 0.53)
**Sex**			
Female	Reference	Reference	Reference
Male	1.42(1.37- 1.47)	1.35(1.32- 1.37)	1.57(1.52- 1.62)
**Race**			
White	Reference	Reference	Reference
Black or African American	0.94(0.89- 0.98)	1.17(1.14- 1.21)	1.15(1.10- 1.20)
Asian	1.14(1.04- 1.24)	1.18(1.11- 1.24)	1.60(1.48- 1.74)
Other	1.28(1.14- 1.45)	1.26(1.18- 1.35)	1.57(1.42- 1.73)
**RUCA Code**			
Urban	Reference	Reference	Reference
Large rural	1.29(1.19- 1.39)	1.08(1.03- 1.14)	1.39(1.30- 1.48)
Small rural	1.41(1.28- 1.55)	1.21(1.12- 1.29)	1.33(1.21- 1.45)
Isolated	1.39(1.22- 1.57)	1.22(1.11- 1.33)	1.35(1.20- 1.51)
**Quarter of diagnosis**			
2020 Q1	Reference	Reference	Reference
2020 Q2	0.70(0.65- 0.75)	0.69(0.65- 0.73)	0.45(0.42- 0.48)
2020 Q3	0.36(0.33- 0.39)	0.49(0.46- 0.52)	0.27(0.25- 0.29)
2020 Q4	0.43(0.40- 0.47)	0.49(0.47- 0.52)	0.26(0.25- 0.28)
2021 Q1	0.39(0.36- 0.42)	0.47(0.45- 0.50)	0.27(0.25- 0.29)
2021 Q2	0.39(0.36- 0.43)	0.48(0.45- 0.51)	0.28(0.26- 0.30)
2021 Q3	0.52(0.46- 0.59)	0.58(0.54- 0.63)	0.34(0.30- 0.38)
**Charlson Comorbidity Index**			
0	Reference	Reference	Reference
1	0.99(0.93- 1.06)	1.10(1.06- 1.14)	1.0(0.94- 1.06)
2	1.01(0.94- 1.08)	1.18(1.14- 1.23)	0.94(0.88- 1.00)
3 or more	1.32(1.26- 1.39)	1.32(1.28- 1.36)	1.01(0.97- 1.06)
**Vitamin D Treatment**			
No	Reference	Reference	Reference
Yes	1.10(1.05- 1.14)	1.78(1.74- 1.84)	1.49(1.44- 1.55)
**BMI**			
Obese	Reference	Reference	Reference
Overweight	0.85(0.81- 0.90)	0.87(0.85- 0.90)	0.71(0.68- 0.74)
Normal weight	0.92(0.87- 0.97)	0.95(0.92- 0.98)	0.62(0.59- 0.65)
Underweight	1.41(1.25- 1.60)	1.03(0.95- 1.12)	0.59(0.51- 0.68)

*Models were also adjusted for data submitter site (not shown here).

Supplementary Table S6: Multivariable Models for Vitamin D and Key Outcomes: Patients with Severe COVID

	Death/Hospice AOR (CI 95%)	Length of Stay > 5 days AOR (CI 95%)	Mechanical Ventilation/ECMO AOR (CI 95%)
**Age category**			
75 and older	Reference	Reference	Reference
65-74	0.58(0.55- 0.60)	1.93(1.80- 2.08)	1.60(1.52- 1.68)
50-64	0.35(0.33- 0.37)	2.77(2.56- 3.00)	1.51(1.44- 1.59)
30-49	0.18(0.16- 0.19)	3.16(2.85- 3.52)	1.18(1.11- 1.25)
18-29	0.09(0.07- 0.10)	2.49(2.10- 2.99)	1.12(1.02- 1.24)
**Sex**			
Female	Reference	Reference	Reference
Male	1.32(1.27- 1.37)	0.97(0.91- 1.02)	1.40(1.35- 1.45)
**Race**			
White	Reference	Reference	Reference
Black or African American	0.88(0.83- 0.92)	1.27(1.17- 1.38)	1.11(1.06- 1.16)
Asian	1.06(0.97- 1.17)	1.18(1.02- 1.38)	1.59(1.45- 1.74)
Other	1.18(1.04- 1.34)	1.18(0.95- 1.48)	1.45(1.30- 1.61)
**RUCA Code**			
Urban	Reference	Reference	Reference
Large rural	1.27(1.18- 1.38)	0.81(0.72- 0.92)	1.36(1.27- 1.47)
Small rural	1.32(1.19- 1.46)	0.80(0.68- 0.94)	1.19(1.08- 1.32)
Isolated	1.25(1.10- 1.43)	0.73(0.60- 0.89)	1.19(1.05- 1.35)
**Quarter of diagnosis**			
2020 Q1	Reference	Reference	Reference
2020 Q2	0.80(0.75- 0.87)	1.06(0.95- 1.19)	0.51(0.47- 0.54)
2020 Q3	0.49(0.44- 0.54)	1.87(1.62- 2.17)	0.36(0.33- 0.39)
2020 Q4	0.59(0.55- 0.64)	1.75(1.56- 1.98)	0.35(0.33- 0.38)
2021 Q1	0.54(0.50- 0.59)	1.64(1.45- 1.86)	0.37(0.34- 0.39)
2021 Q2	0.56(0.50- 0.61)	1.45(1.25- 1.69)	0.37(0.34- 0.41)
2021 Q3	0.72(0.63- 0.82)	1.98(1.57- 2.52)	0.42(0.37- 0.48)
**Charlson Comorbidity Index**			
0	Reference	Reference	Reference
1	0.95(0.89- 1.02)	0.94(0.84- 1.04)	0.93(0.88- 1.00)
2	0.97(0.90- 1.04)	0.98(0.87- 1.11)	0.84(0.78- 0.90)
3 or more	1.18(1.12- 1.25)	0.74(0.68- 0.80)	0.83(0.79- 0.87)
**Vitamin D Treatment**			
No	Reference	Reference	Reference
Yes	0.90(0.86- 0.94)	2.03(1.87- 2.21)	1.16(1.12- 1.21)

**BMI**			
Obese	Reference	Reference	Reference
Overweight	0.89(0.84- 0.94)	1.0(0.91- 1.09)	0.73(0.69- 0.76)
Normal weight	0.93(0.87- 0.98)	0.86(0.78- 0.94)	0.60(0.57- 0.63)
Underweight	1.39(1.21- 1.58)	0.62(0.51- 0.75)	0.53(0.45- 0.62)

*Models were also adjusted for study site (not shown here).

Supplementary Table S7. Key variables and concept definitions

<table>
<tr><td>Variable</td><td></td><td>Concepts and Logic</td></tr>
<tr><td></td><td colspan="2">Primary Cohort Definitions</td></tr>
<tr><td>SARS-CoV-2 Infection</td><td></td><td>
<u>Definition</u>: Positive Lab measurements (PCR, Antigen or Antibody) or COVID diagnosis.
<u>Concept Sets used</u>:

ATLAS SARS-CoV-2 rt-PCR and AG

Latest Codeset Id: 651620200 (most recent version)
Description: Includes 55 Concepts (Measurements), characterizing a positive PCR or Antigen test.

Atlas #818 [N3C] CovidAntibody retry

Latest Codeset Id: 45478367 (most recent version)
Description: Includes 24 Concepts (Measurements), characterizing a positive Antibody test.

N3C Covid diagnosis

Latest Codeset Id: 35486128
Description: Corresponds to ICD10CM Code: U07.1 (condition). Includes 1 Concept (Disease caused by 2019-nCoV - Concept Id: 840539006).

ResultPos

Latest Codeset Id: 400691529
Description: different ways that sites report a positive value for a lab test (e.g. Positive, Detected, Reactive, Presumptive Positive etc.)

</td></tr>
<tr><td>Hospitalization</td><td></td><td>
<u>Visit Concept IDs and Names:</u>
262 Emergency Room and Inpatient Visit
8717 Inpatient Hospital
9201 Inpatient Visit
581379 Inpatient Critical Care Facility
32037 Intensive care
581385 Observation room
</td></tr>
</table>

		**Logic:**<br><br>No concept sets were used in this definition. Includes patients with a visit start date within 28 days following their first COVID diagnosis and a length of stay greater than 0.
	Outcomes	
Death or referral to hospice		**Logic:**<br><br>No concepts or concept sets were used. Patients were flagged as deceased if a valid entry with a date was included in the death table, which is part of the OMOP data model used in the Enclave. Patients were flagged as referred to hospice if "discharge_to_concept_name" in the visit occurrence table contained "hospice."
Length of stay > 5 days		Discharge date or death date/referral to hospice date – admission date
Mechanical Ventilation		**Concept Set Names (latest codeset ID):**<br><br>Invasive Mechanical Ventilation (179437741)<br><br>**Logic:**<br><br>Includes patients associated with any procedure codes in the concept sets listed that had a procedure date during their COVID hospitalization.
ECMO		**Concept Set Names (latest codeset ID):**<br><br>Kostka – ECMO (415149730)<br><br>**Logic:**<br><br>Includes patients associated with any procedure codes in the concept sets listed that had a procedure date during their initial post-COVID hospitalization.
	Key Exposures	
Rurality		**Logic:**<br><br>Patients were included if they had a 5-digit ZIP Code, which could be mapped to RUCA Codes through an external dataset maintained by the USDA Economic Research Services: https://www.ers.usda.gov/data-products/rural-urban-commuting-area-codes/. This

		dataset is available in N3C here: [EXTDATASET-59] RUCA Rural-Urban Commuting Area Codes 1. Patients were mapped to four categories based on this ZIP Code crosswalk as follows based on primary RUCA Code designation:<br><br>1: Metropolitan<br>2: Large rural<br>3: Small rural<br>4: Isolated rural
BMI		Reported BMI from measurement table. Additional information obtained for BMI categories from observation table.
Vitamin D		Drug concept IDs: 40167891, 42708308, 40241647, 42708755, 42874153, 37498643, 40239082, 42901711, 40237634, 42707662, 42707512, 43012510, 40239423, 19099870, 42708126, 19036526, 40167890, 45775844, 40244060, 1718579, 42874161, 40230514, 43012672, 40167886, 43526061, 40244059, 44818394, 19079866, 40167885, 19008521, 40224268, 43012511, 19133347, 40167963, 46233867, 19129422, 19133346, 43526044, 19135253, 42629276, 43011952, 19036525, 19036527, 19132783, 19008496, 40229616, 40238790, 37498645, 19133815, 43560507, 19131640, 42708309, 36249586, 42800447, 42708310, 44785642, 1510303, 40167888, 19075302, 19133754, 40240596, 44818395, 19133751, 44507486, 42707347, 43526056, 19101536, 19126425, 40224277, 40167992, 40224241, 19133752, 42898767, 19121187, 19125401, 43012673, 19132795, 40224265, 40240599, 42708125, 1511290, 1594025, 19009405, 19041926, 42629279, 19132782, 35603968, 40163055, 40175613, 40239689, 40167040, 40173606, 40222052, 46221643, 19074587, 19121193, 19009407, 35603108, 40167968, 42709324, 43560514, 43012580, 46233733, 44507487, 40235689, 44785197, 19133749, 40235683, 19045071, 40166212, 19132804, 42707306, 19095181, 40224257, 42708127, 42708124, 19036366, 19135161, 19045050, 19058738, 793944, 19036426, 42898768, 40166213, 46233870, 19095165, 19121201, 19095164, 40241698, 40173610, 40173607, 19074527, 35201021, 19109298, 40242471, 42901643, 19131706, 19095246, 40019687, 42800467, 19132797, 40237701, 19133750, 1511291, 43255094, 35604410, 40240598, 40236773, 19076926, 40168350, 40239710, 19135544, 40237674, 19133753, 19016422, 40235565, 19061366, 40242473, 19045048, 42707307, 1593123, 42873790, 19125845, 40224271, 40173609, 40227836, 40171690, 43531768, 19112817, 43560134, 19095225, 19125897, 19131348, 40025589, 40183115, 42629565, 40125338, 40242469, 19045045, 40235688, 40020053, 21129466, 40169625, 40237731, 19105858, 40169627, 1592263, 40168717, 19095248, 19059823, 19057624, 42799410, 43012671, 19108285, 40236808, 42707354, 40165345, 19009436

	Comorbid Conditions used to calculate Charlson Comorbidity Index	
Severe cardiovascular event (Congestive heart failure and myocardial infarction)		Concept Set Names (latest codeset ID):<br>Charlson - CHF (359043664)<br>Charlson - MI (259495957)<br><br>Logic:<br>Includes patients associated with any condition codes in the concept sets listed with an occurrence date before the earliest covid diagnosis.
Peripheral vascular diseases		Concept Set Names (latest codeset ID):<br>Charlson - PVD (376881697)<br><br>Logic:<br>Includes patients associated with any condition codes in the concept sets listed with an occurrence date before the earliest covid diagnosis.
Stroke		Concept Set Names (latest codeset ID):<br>Charlson - Stroke (652711186)<br><br>Logic:<br>Includes patients associated with any condition codes in the concept sets listed with an occurrence date before the earliest covid diagnosis.
Dementia		Concept Set Names (latest codeset ID):<br>Charlson - Dementia (78746470)<br><br>Logic:<br>Includes patients associated with any condition codes in the concept sets listed with an occurrence date before the earliest covid diagnosis.
Pulmonary Diseases		Concept Set Names (latest codeset ID):

		Charlson - Pulmonary (514953976)<br><br><br>**Logic:**<br><br>Includes patients associated with any condition codes in the concept sets listed with an occurrence date before the earliest covid diagnosis.
Rheumatic Diseases		**Concept Set Names (latest codeset ID):**<br><br>Charlson - Rheumatic (765004404)<br><br><br>**Logic:**<br><br>For all RA patients this value is set to 1.
Peptic ulcer diseases		**Concept Set Names (latest codeset ID):**<br><br>Charlson - PUD (510748896)<br><br><br>**Logic:**<br><br>Includes patients associated with any condition codes in the concept sets listed with an occurrence date before the earliest covid diagnosis.
Liver diseases (mild and severe liver diseases)		**Concept Set Names (latest codeset ID):**<br><br>Charlson - LiverMild (494981955)<br><br>Charlson - LiverSevere (248333963)<br><br><br>**Logic:**<br><br>Includes patients associated with any condition codes in the concept sets listed with an occurrence date before the earliest covid diagnosis.
Diabetes mellitus (diabetes mellitus and diabetes mellitus with complications)		**Concept Set Names (latest codeset ID):**<br><br>Charlson - DM (719585646)<br><br>Charlson - DMcx (403438288)<br><br><br>**Logic:**<br><br>Includes patients associated with any condition codes in the concept sets listed with an occurrence date before the earliest covid diagnosis.

Renal diseases		<u>Concept Set Names (latest codeset ID):</u><br><br>Charlson - Renal (220495690)<br><br><u>Logic:</u><br><br>Includes patients associated with any condition codes in the concept sets listed with an occurrence date before the earliest covid diagnosis.
Cancer (metastatic and non-metastatic)		<u>Concept Set Names (latest codeset ID):</u><br><br>Charlson - Cancer (535274723)<br><br>Charlson - Mets (378462283)<br><br><u>Logic:</u><br><br>Includes patients associated with any condition codes in the concept sets listed with an occurrence date before the earliest covid diagnosis.
		Additional Comorbid Conditions (Non-Charlson Comorbidity Index)
Hypertension		<u>Concept Set Names (latest codeset ID):</u><br><br>Hypertension_Elixhauser_UC (779214702)<br><br><u>Logic:</u><br><br>Includes patients associated with any condition codes in the concept sets listed with an occurrence date before the earliest covid diagnosis.
